# PPAR*γ* in Kidney Physiology and Pathophysiology

**DOI:** 10.1155/2008/183108

**Published:** 2009-03-10

**Authors:** Éva Kiss-Tóth, Tamás Rőszer

**Affiliations:** ^1^Department of Biochemistry and Molecular Biology, Medical and Health Science Center, University of Debrecen, Nagyerdei krt. 98, 4012 Debrecen, Hungary; ^2^Research Group of Apoptosis and Genomics, Hungarian Academy of Sciences, Nagyerdei krt. 98, 4012 Debrecen, Hungary

## Abstract

Involvement of the nuclear receptor peroxisome proliferator-activated receptor gamma (PPAR*γ*) in kidney physiology has been explored recently. Synthetic PPAR*γ* ligands can ameliorate the diabetic kidney disease through different mechanisms, involving inhibition of mesangial cell growth, reduction of mesangial matrix, and cytokine production of glomerular cells as well as promoting endothelial cell survival within the kidney glomeruli. Activation of PPAR*γ* has additional profibrotic consequences, which can contribute to wound healing in diabetic glomerulonephritis. Beside many beneficial effects, PPAR*γ* activation, however, can lead to severe water retention, a common side effect of thiazolidinedione therapy. This unwanted effect is due to the activation of PPAR*γ* in the mesonephric distal collecting system, where PPAR*γ* positively regulates sodium and water resorbtion leading to the expansion of interstitial fluid volume. Recent studies indicate that PPAR*γ* is also involved in the normal kidney development, renal lipid metabolism, and activation of the renin-angiotensin system. In this paper, we give a synopsis of the current knowledge on PPAR*γ* functions in kidney phyisology and pathophysiology.

## 1. INTRODUCTION

The nuclear receptor peroxisome proliferator-activated receptor gamma (PPAR*γ*) regulates transcription of various genes involved in lipid uptake, fatty acid metabolism, and glucose homeostasis [1], therefore, the modulation of PPAR*γ* action is of intense interests in the medication of insulin resistance and related metabolic disorders [2–7]. Pharmacological activation of PPAR*γ* facilitates the glucose and free fatty acid flux from striated muscle fibers to adipocytes and reduces liver gluconeogenesis by which PPAR*γ* exerts antidiabetic benefits [1, 5, 6]. PPAR*γ* signaling can also influence the expression of insulin-dependent glucose transport (GLUT) proteins [8], and can induce the production of hormone-like substances in adipose cells (e.g., resistin and adipokines) promoting insulin responsiveness [1]. Recent studies indicate that impaired insulin sensitivity of skeletal muscle and white adipose tissue can be a consequence of a chronic subclinical inflammation [2–6]. Activation of PPAR*γ* in macrophages has anti-inflammatory effects, by which PPAR*γ* ligands can reduce the local low-grade inflammation and consequent insulin resistance of muscle and adipose tissues [5, 6]. Thiazolidinediones (TZDs), synthetic ligands of PPAR*γ* are clinically proven insulin sensitizers with antiinflammatory benefits. Nowadays, TZD therapy is a widely used medication strategy of type 2 diabetes and related diseases [5, 6].

Beside beneficial effects of TZD therapy in insulin resistance, edema and water retention also frequently occurs as secondary effects of PPAR*γ* activation [9, 10]. The understanding of TZD side effects highly facilitated the basic research on PPAR*γ* and kidney physiology. As a result, several fundamental findings on the involvement of PPAR*γ* in fluid homeostasis have been explored in the recent years [1, 10–18]. These findings indicate that PPAR*γ* is involved in the regulation of sodium and water resorbtion of the distal collecting ducts of the kidney which explains the unwanted TZD effects on interstitial fluid volume regulation [9, 17, 18]. Due to the anti-inflammatory roles of PPAR*γ* activation, the receptor is involved in the attenuation of glomerulonephritis, which is also a potent therapeutic value of TZDs [10–16].

Many other roles are also attributed to PPAR*γ* in normal kidney development, lipid metabolism, and endocrine functions [19]. In this paper, we give a synopsis of PPAR*γ* actions as well as the PPAR*γ*-independent effects of synthetic PPAR*γ* ligands in kidney phyisology and pathophysiology.

## 2. PPAR*γ* IN THE FILTRATION UNITS OF THE KIDNEY

### 2.1. Diabetic kidney disease is coupled to impaired mesangial cell functions

**Figure 1:**
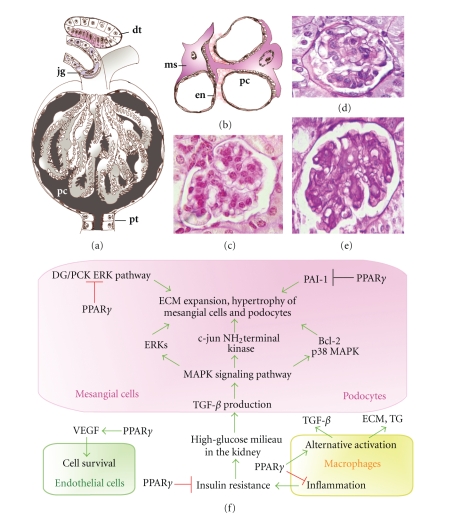
Roles of PPAR*γ* in the filtration units of the kidney. The kidney capsules (a) contain the glomerular capillaries covered with podocytes (pc). In the wall of the afferent arterioles, modified smooth muscle cells form the juxtaglomerular system (jg). The filtrated urine is guided to the proximal tubules (pt). The distal tubules (dt) can return to the cortical kidney capsules and their epithelial layers serve as a chemosensory region, the macula densa (labeled with red). (b) PPAR*γ* activation affects either podocyte (pc), mesangial cell (ms), or endothel cell (en) functions. (c) Periodic acid-Schiff (PAS) stained sections of a normal kidney capsule in mouse. (d) Glomerulonephritis in high-fat diet fed mouse and (e) type 2 diabetic (db/db) mouse, showing intensive PAS staining of the expanded mesangial matrix, thickening of glomerular walls, and enlargement of kidney capsules. (f) Summary of PPAR*γ*-mediated cellular events in mesangial cells, podocytes, kidney macrophages, and glomerular endothel cells.

In the latest years, several articles explored the conneciton of PPAR*γ* and the impaired function of the kidney filtration units in diabetic kidney disease [10, 12–15]. More than 30% of patients with juvenile or maturity onset diabetes mellitus develop clinically evident diabetic glomerulopathy within 10–20 years of the diabetes onset [16, 20]. After years of poor glycemic control, the structure of the glomerular walls get scarred and permeability changes can develop which are core features of the diabetic glomerulosclerosis or glomerulonephritis [20]. The disease is characterized by the strong accumulation of extracellular matrix proteins (Figure 1) and deposition of type IV collagen in the glomerular mesangium leading to the expansion of mesangial matrix and glomerular size [10, 12–16, 21, 22]. Elevated glomerular size can manifest in kidney hypertrophy [20]. Alterations of the glomerular morphology lead to fluid filtration deficits, albuminuria, glucosuria, and finally reduction of glomerular filtration [21–30].

Glomerular mesangial cells have a central role in the development of diabetic glomerulonephritis (Figure 1(b)), since these cells can overproduce the extracellular matrix proteins of the glomerular mesangium in response to chronic hyperglycemia [11–13, 21].

### 2.2. Effects of PPAR*γ* activation in mesangial cells

Activation of PPAR*γ* as well as PPAR*α* in mesangial cells can attenuate the overproduction of the mesangial matrix (Figure 1(f)), as it has been described in animal models of diabetic nephropathy [21, 22]. Diabetes in apolipoprotein-E (ApoE)-deficient mice is associated with a significant accumulation of extracellular matrix proteins and increased immunostaining for collagen IV in the glomerular compartments (Figures 1(d), 1(e)). Treatment with rosiglitazone results in a significant reduction in collagen IV deposition [21]. In Otsuka Long-Evans Tokushima Fatty (OLETF), type 2 diabetic rats glomerular hypertrophy correlates well with the expression of large quantities of the Bcl-2 protein, an apoptosis-suppressing molecule in the mesangial cells [22]. This finding suggests that persistent proliferation and prolonged survival of the mesangial cells can also contribute to the supernormal matrix secretion in glomerulopathy. The gene encoding Bcl-2 has a PPAR response element by which PPAR*γ* can increase Bcl-2 mRNA transcription. However, some reports have indicated that TZD treatment can decrease the level of Bcl-2 and induce apoptosis independently of PPAR*γ* [22].

TZDs cannot only reduce glomerular cross-sectional area and the mesangial matrix size as well as collagen IV synthesis but also enhance the tumor growth factor beta-1 (TGF-*β*1) positive staining areas in the kidney of OLETF rats [22]. TGF-*β* seems to be a central molecule in the PPAR agonist action [10, 12, 22]. This growth factor activates several intracellular signal transduction systems involved in the regulation of the extracellular matrix biosynthesis (Figure 1(f)), including mitogen-activated protein kinases (MAPKs), the extracellular signal-regulated kinases (ERKs), the c-jun NH_2_-terminal kinases, diacylglycerol/protein kinase C extracellular signal-regulated kinase pathway, and the p38 MAPK [23–30]. PPAR*γ* agonists besides their anti-inflammatory effect can inhibit TGF-*β* expression leading to a repression in glomerular proliferation [16, 22, 30]. PPAR*γ* also has a direct effect on key extracellular matrix regulators as plasminogen activator inhibitor-1 (PAI-1). PAI-1 is a member of the serine protease inhibitor superfamily and it can inhibit proteolysis of the extracellular matrix, leading to matrix accumulation and sclerosis. PPAR*γ* agonists may inhibit PAI-1 transcription by antagonizing the activities of activator protein-1 (AP-1) and nuclear factor *κ*B [23–31].

The presence of TGF-*β*1 in the mesangial cells refers to a mechanism by which high-glucose milieu induces inflammatory and profibrotic cytokine production in glomerular cells (Figure 1(f)). In diabetic nephropathy, mesangial cells as well as podocytes and interstitial cells can secrete monocyte chemoattractant protein-1 (MCP-1) and TGF-*β*1 which may initiate macrophage infiltration into the kidney [10, 12–15]. The number of infiltrated machrophages is being increased both in the glomeruli and the renal interstitium with the development of diabetic kidney disease in OLETF rats. TZDs have an anti-inflammatory effect in the peripheral tissues, therefore treatment with pioglitazone or rosiglitazone decreases macrophage infiltration of the kidney [19, 22, 30]. MCP-1 can also influence the alternative macrophage activation. Alternatively activated macrophage release factors such as IL-1ra/IL-1F3, IL-10, and TGF-*β* [31–33]. TGF-*β* functions indirectly to promote extracellular matrix building by inducing nearby kidney fibroblasts to produce matrix components [34]. The alternatively activated macrophages themselves produce extracellular matrix components, as fibronectin and a cross-linking enzyme transglutaminase (Figure 1(f)), as well as osteopontin, which is involved in cell adhesion to the matrix [32, 35]. The molecules secreted by the alternatively activated macrophages can promote wound repair due to their anti-inflammatory, fibrotic, proliferative, and angiogenic activities [32–35].

### 2.3. Role of PPAR*γ* in podocytes and capillaries in glomerulonephritis

Podocyte injury is also among the primary events in early development of the glomerulosclerosis [33, 36]. A decrease in podocyte number in type 2 diabetic Pima Indians correlates closely with those patients who have microalbuminuria, the earliest manifestation of diabetic nephropathy [37]. High-glucose treatment or the epithelial cell toxin puromycin aminonucleosid (PAN) supplementation induces podocyte injury and PPAR*γ* upregulation in podocyte culture [37]. This increase of PPAR*γ* is counterregulatory and might promote podocyte healing and repair. Pioglitazone treatment of podocytes can inhibit expression or phosphorylation of cell proliferation and antiapoptotic proteins (e.g., p27^Kip1^, p42 MAPK, Bcl-2) which can be one major molecular mechanism behind the therapeutic potential of TZDs on high glucose-induced hypertrophy of podocytes [22, 23].

Microangiopathy of glomerular capillaries is also a hallmark of the diabetic nephropathy [10, 12–15, 33]. Endothelial growth and survival are regulated by two factors, vascular endothelial growth factor (VEGF) and angioprotein which are also expressed by podocytes (Figure 1(f)). PPAR*γ* agonists can protect glomerular capillaries against injury both by increasing podocyte VEGF expression and by decreasing Aglp4 [38]. TZDs, therefore, can prevent angiopathy of the capillaries in the glomeruli, one causing event of progressive kidney disease.

## 3. PPAR*γ* IN THE DISTAL COLLECTING SYSTEM

### 3.1. Expression of PPAR*γ* in the nephron ducts

**Figure 2:**
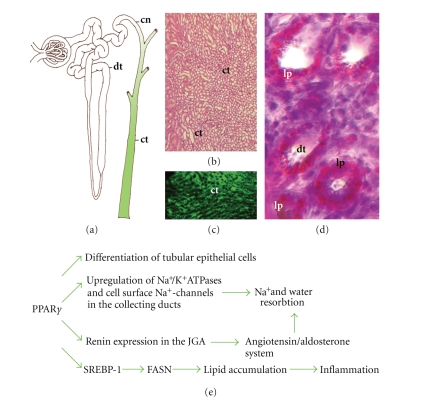
Roles of PPAR*γ* in the collecting system of the kidney. (a) Expression of PPAR*γ* is confined to the distal collecting system (labeled with green) including connective tubules (cn) and collective ducts (ct). (b) Hematoxylin and esoin stained cross-sections of the kidney medulla showing numerous collective ducts (ct). (c) Fluorescent PPAR*γ* immunostaining in the same region of the kidney. (d) Oil red-O stained sections of the distal tubules (dt) showing severe lipid accumulation in type 2 diabetic (db/db) mice. (e) Summary of PPAR*γ* functions in the collective system.

Under physiological conditions, PPAR*γ* is dominantly expressed in the collecting system of the mammalian urinary tract, including connective renal tubules and collecting ducts (Figure 2(a)). PPAR*γ* is abundant in the inner renal medulla (Figures 2(b), 2(c)) and localized to the epithelial layer starting from medullary collecting ducts to the urothelium of the ureter and the bladder [39–41]. PPAR*γ* also occurs in renal medullary interstitial cells [39]. The PPAR*γ* partner RXR*α* has a complimentary distribution in the collecting ducts [42]. The connective tubules and collective ducts are parts of the distal collecting system, where hormone-regulated ion exchange and water resorbtion takes place and provides the balance of interstitial fluid volume (Figure 2(e)). If aldosterone is present, sodium is resorbed and potassium is secreted. Sodium transport is followed by passive water resorbtion, therefore, this mechanism regulates the total electrolite and water volume in the body [43]. The epithelium of the collecting ducts is responsive to antidiuretic hormone. If the hormone is present, the epithelia becomes permeable to water. The distal collecting system is, therefore, a major site of fluid volume regulation.

### 3.2. Embryology and phylogenetic homologies of PPAR*γ* expressing collecting ducts

In mammals, the development of the kidney collecting system differs from the other excretory parts of the kidney [44]. Collecting ducts and tubules are formed by the ureteric bud, which is an outgrowth of the dorsomedial wall of the mesonephric duct. The proliferating mesonephric bud penetrates the developing metanephric tissues and dilates forming the primitive renal pelvis and calyces. The further subdivisions of the calyces form the presumptive collecting ducts [44]. According to the recent literature, PPAR*γ* expression is mainly confined to the collecting system of the kidney [39, 41, 45–55], which has a mesonephric origin (Figure 2(a)). A lower expression of PPAR*γ*1 in the proximal tubules, which are derived from the metanephric tissue, has been indicated in the rat kidney [56] while in mesangial cells and podocytes of the kidney capsules PPAR*γ* is upregulated only under pathological conditions as chronic hyperglycemia or glomerulonephritis [10, 12–15]. The distribution pattern of PPAR*γ* suggests that PPAR*γ* may have been coupled to the mesonpehros in the vertebrate phylogeny. Supporting this possibility, the kidney of teleost fishes, which is a functioning mesonephros and a phylogenic homolog of the mammalian collecting system, contains all of the three PPAR isoforms [57–59]. Like their mammalian homologs, fish PPARs bind to a variety of natural PPAR response elements (PPREs) present in the promoters of mammalian or piscine genes.

### 3.3. Role of PPAR*γ* in the balance of fluid homeostasis

As its distribution pattern suggests, the clinically most relevant function of PPAR*γ* is the modulation of electrolyte and water resorbtion [17, 18, 41, 60]. Edema and fluid retention are common and serious side effects of TZD therapy, which are due to supernormal sodium resorbtion and consequent interstitial fluid volume expansion [9, 32]. Since PPAR*γ* is a significant target for TZDs and it is predominantly expressed in the collecting ducts, critical sites for the control of fluid metabolism, its possible involvement in fluid metabolism has been recently elucidated. PPAR*γ* activation can modulate sodium resorbtion through the stimulation of epthelial sodium channels and the Na^+^/K^+^-ATPase system [41, 60]. Additionally, TZDs can ditsurb the renin-angiotensin-aldosterone system also (Figure 2(e)). In human collecting duct cell culture PPAR*γ* activation enhances the expression of cell surface epithelial sodium channels which can facilitate the sodium resorbtion ability of the tubular cells [41]. The role of PPAR*γ* in the regulation of sodium resorbtion has been also confirmed by studies carried out on mice with collecting duct-specific ablation of PPAR*γ* [17, 18]. These studies show a critical role for PPAR*γ* in systemic fluid retention through the regulation of renal sodium transport, and that the adverse effects of TZD in fluid metabolism are indeed PPAR*γ*-dependent. A gene encoding for the gamma subunit of the epithelial sodium channel has been identified as a critical PPAR*γ* target gene in the control of electrolyte and water resorbtion of the collecting ducts (Figure 2(e)).

### 3.4. Proliferation and metabolism of kidney epithelia and effects of PPAR*γ*

PPAR*γ* has some additional functions in the collecting system of the kideny. During embryogenesis, the expression of PPAR*γ* in urothelium [41, 46, 55] suggests its possible involvement in the urothelial proliferation and differentiation. In cultured rat kidney epithelial cells, both troglitazone and 15d-PGJ_2_ significantly inhibit cell proliferation and dramatically alter cell shape by induction of cell process formation [19, 41]. TZDs or PPAR*γ* overexpression induces the Klotho gene expression in mouse kidneys and renal epithelial cell culture promoting insulin sensitivity and reducing cellular aging [46].

The PPAR*γ* ligand TZDs alter not only cellular growth and survival but also metabolic processes of the kidney collecting duct epithelia including carbohydrate, lipid metabolism, and albumine transport [19, 47]. TZDs can activate PPAR*γ*-regulated genes as well as P-ERK and AMP-activated protein kinase pathways which modulate gluconeogenesis, cellular acidosis, glutamine metabolism, and ammoniagenesis of porcine tubular cells [19]. It is possible that modulation of kidney carbohydrate metabolism by TZDs has a beneficial role in the glycemic control [19]. Interestingly some in vitro studies with kidney epithelial cells of the opossum have revealed that TZD affects protein handling of tubular epithelia also [47]. Rosiglitazone, ciglitazone, and troglitazone can inhibit the uptake of FITC-labeled albumin by tubular epithelial cells in a dose-dependent manner without any cytotoxic effect. Unexpectedly, in tubular cells overexpressing PPAR*γ* or in cells treated with the PPAR*γ* antagonist GW9662, albumin handling cannot be affected. Similarly, the PPAR*γ* ligand 15d-PGJ2, which is structurally unrelated to TZDs, has no effect on albumin uptake [47]. Albumin handling of tubular cells can be, therefore, affected by TZDs independently from PPAR*γ*. Effects of TZDs on tubular protein uptake, however, can be physiologically less relevant than the benefits of TZD administration on glomerular functions which conseqeuntly reduce albuminuria.

PPAR*γ* is also involved in the renal lipid metabolism (Figures 2(d), 2(e)). Abnormal renal lipid synthesis plays a role in the pathogenesis of diabetic nepropathy [48]. Renal lipid deposits in glomerulosclerosis have been mentioned even in the first description of the diabetic kidney alterations by Kimmelstiel and Wilson in 1936 [49]. Lipid deposits are present in the kidney of diabetic humans as well as of diabetes model rodents [48–55, 61, 62]. In diabetic animals upregulation of kidney SREBP-1, the key enzyme of fatty acid synthesis can lead to the renal accumulation of lipids as well as mesangial matrix expansion and kidney hypertrophy [51, 52]. Elevated levels of plasma lipids also can contribute to renal fat deposition and facilitate the development of glomerulosclerosis [53]. High glucose concentration can also increase SREBP-1 expression in cultured rat mesangial cells, suggesting that impaired glycemic control can disturb renal lipid metabolism through altered SREBP-1 gene expression, which is regulated by PPAR*γ* [51]. It is possible that the transcriptional activity of PPAR*γ* in the duct cells is upregulated by insulin and C-protein, a protein fragment of proinsulin [54]. Both insulin and C-peptide can induce a concentration-dependent activation of PPAR*γ* and both agents can augment the TZD-stimulated PPAR*γ* activity giving the possibility that hyperinsulinemia in type 2 diabetes can augment PPAR*γ* as well as PPAR*γ*-regulated SREBP-1 gene functions.

Renal lipid accumulation, however, not only is a consequence of the hyperglycemia or dyslipidemia but also can predispose or provoke glomerulonephritis. Recent in vitro studies suggest that low-density lipoproteins and very low-density lipoproteins induce upregulation of growth factors, TGF-*β*, and matrix proteins in cultured renal mesangial and tubular cells [55, 61]. This direct effect of lipids on gene expression of kidney cells can initiate the development of mesangial matrix expansion which is a hallmark of glomerulonephritic syndrome. In mice with upregulated SREBP-1 expression, the signs of glomerulonephritis as albuminuria, renal cholesterol, and triglyceride deposits occur without changes in glucose homeostasis or serum lipid levels [51]. In these SREBP-1 transgenic mice, the elevated renal lipid content is coupled with increased TGF-*β* and vascular endothelial growth factor (VEGF) expression [51]. VEGF plays a pivotal role in the pathogenesis of glomerulosclerosis [63]. PPAR*γ* haploinsufficiency as well as Pro12Ala (P12A) allele polymorphism of PPAR*γ* has a protective role in the development of diabetic nephropathy [64]. In mice with heterozygous PPAR*γ* mutation, high-fat diet results in a less severe nephropathy and lipid depositions than in wild type animals [45].

## 4. PPAR*γ* FUNCTION IN THE JUXTAGLOMERULAR APPARATUS

Kidney is not only an excretory organ but also serves endocrine functions by the secretion of renin, a 37 kDa protein hormone produced by the juxtaglomerular cells. Juxtaglomerular cells are modified smooth muscle cells in the media of the afferent arteriole adjacent to the renal capsule (Figure 1(a)). Renin acts on a plasma protein called angiotensinogen, producing an inactive decapeptide, the angiotensin I. This substance as a result of the action of a converting enzyme present in high concentration in lung endothelial cells, becoming an octapeptide called angiotensin II. Angiotensin II enhances the secretion of aldosterone in the adrenal gland [65, 66]. The main targets of aldosterone are the distal tubules, where it can regulate sodium reabsorption (Figure 2(e)).

Human renin gene enhancer is modulated by PPAR*γ* activation [67, 68]. In human renin-producing cell line CaLu-6, endogenous or pharmacological PPAR*γ* agonists (unsaturated fatty acids and TZDs) can stimulate renin mRNA transcription [67, 68].

Although renin production is facilitated by PPAR*γ* activation, the hypertensive effects of angiotensin II can be attenuated by TZDs [69–71]. In addition to its role in controlling water and salt homeostasis, the inhibition of the renin-angiotensin system reduces the incidence of type 2 diabetes in patients with hypertension or congestive heart failure and also reduces the risk of nephropathy in diabetic patients [71]. The mechanisms underlying these protective effects appear to be complex and may involve an improvement of both insulin sensitivity and insulin secretion. Recent works suggest that aldosterone and mineralocorticoid receptors regulate PPAR*γ* expression [72, 73]. Aldosterone as well as angiotensin receptor blockers appear to induce PPAR*γ* activity in the adipose tissue, which could explain the protective effect of the renin-angiotensin system inhibition against the development of type 2 diabetes [71]. It is unlikely, however, that the favorable effects of TZDs on diabetic nephropathy would be related to a dierct effect on the renin-angiotensin system [74].

## 5. CYTOTOXIC EFFECTS OF PPAR*γ* LIGANDS ON TUBULAR EPITHELIAL CELLS

Synthetic PPAR*γ* ligands are widely used drugs for the treatment of insulin resistance. There is an evidence that these drugs have beneficial effects on the improvement of metabolic parameters as proteinuria in type 2 diabetes, however, some severe metabolic secondary effects have been recognized [75–77].

Increasing number of synthetic PPAR*γ* ligands is commercially available today (e.g., troglitazone, rosiglitazone, pioglitazone, ciglitazone, muraglitazar) for treatment of type 2 diabetes complications. Many reports have described the side effects of them including antiproliferative and apoptotic actions in cultures of renal proximal tubular cells [78], mesangial cells [79], and interstitial fibroblasts [80]. Ciglitazone has a direct necrotic effect on renal proximal tubular cells at a concentration range similar to its therapeutical plasma levels. Interestingly, these cytotoxic effects are not universal for all PPAR*γ* agonists because pioglitazone is not cytotoxic in the same cell lines [81]. Although renoprotective effects of dual PPAR*α* and PPAR*γ* activation have been reported in type 2 diabetic animals [82], muraglitazar (a PPAR*α*/*γ* dual agonist) can induce multifocal urothelial necrosis and proliferation in young male rats which is thought to be provoked by muraglitazar-associated changes in urine composition [83].

## 6. SUMMARY

PPAR*γ* agonists have many beneficial effects combined with their independent antiatherosclerotic actions and their important effects on dyslipidemia and insulin resistance in the medication of kidney disease coupled to diabetes [10, 12–15, 21, 82]. Activation of PPAR*γ* attenuates diabetic glomerulonephritis due to its anti-inflammatory and profibrotic effects [32–35]. PPAR*γ* and PPAR*α* have similar antidiabetic and renoprotective effects, therefore administration of PPAR*α* or PPAR*α*/*γ* dual agonists may be also useful for the prevention of kidney complications of type 1 as well as type 2 diabetes mellitus [13, 21, 82]. On the other hand, PPAR*γ* signaling can facilitate lipid accumulation or induce a direct necrotic cell death of tubular epithelial cells, therefore synthetic PPAR*γ* ligands, especially TZDs should be used with a great foresight in the medication of insulin-resistant diabetes mellitus [1, 48–54].

The most recently discovered role of PPAR*γ* in the positive regulation of salt and water resorbtion have elucidated the pathomechanism of water retention and edema in patients treated with TZDs, the widely used PPAR*γ* agonists [17, 18]. Edema and fluid retention can be fatal side effects of TZDs, which can be attenuated by the combination of TZD therapy with diuretics [9]. The selective PPAR modulator (SPPARM) approach has also been proposed as a method to avoid unwanted complications of PPAR*γ* ligands [9].

Some comparative data suggest that PPAR*γ* is coupled to the mesonephric parts of the vertebrate kidney, therefore the involvement of PPAR*γ* in the intesrtitial fluid volume regulation can be an ancient and evolutionarily conserved role [56–58]. Some other components of renal PPAR*γ* activation, including the function of PPAR*γ* in the moduation of renal endocrine functions, are still undefined [67, 74] indicating the timeliness of future research in the field of PPAR*γ* and kidney physiology.
